# Metagenome Mining Reveals Hidden Genomic Diversity of Pelagimyophages in Aquatic Environments

**DOI:** 10.1128/mSystems.00905-19

**Published:** 2020-02-18

**Authors:** Asier Zaragoza-Solas, Francisco Rodriguez-Valera, Mario López-Pérez

**Affiliations:** a Evolutionary Genomics Group, División de Microbiología, Universidad Miguel Hernández, Alicante, Spain; b Laboratory for Theoretical and Computer Research on Biological Macromolecules and Genomes, Moscow Institute of Physics and Technology, Dolgoprudny, Russia; University of Vienna

**Keywords:** Fonsibacter, pelagiphages, SAR11, genome-resolved metagenomics, myophages

## Abstract

The SAR11 clade is one of the most abundant bacterioplankton groups in surface waters of most of the oceans and lakes. However, only 15 SAR11 phages have been isolated thus far, and only one of them belongs to the *Myoviridae* family (pelagimyophages). Here, we have analyzed 26 sequences of myophages that putatively infect the SAR11 clade. They have been retrieved by mining ca. 45 Gbp aquatic assembled cellular metagenomes and viromes. Most of the myophages were obtained from the cellular fraction (0.2 μm), indicating a bias against this type of virus in viromes. We have found the first myophages that putatively infect *Candidatus* Fonsibacter (freshwater SAR11) and another group putatively infecting bathypelagic SAR11 phylogroup Ic. The genomes have similar sizes and maintain overall synteny in spite of low average nucleotide identity values, revealing high similarity to marine cyanomyophages. Pelagimyophages recruited metagenomic reads widely from several locations but always much more from cellular metagenomes than from viromes, opposite to what happens with pelagipodophages. Comparing the genomes resulted in the identification of a hypervariable island that is related to host recognition. Interestingly, some genes in these islands could be related to host cell wall synthesis and coinfection avoidance. A cluster of curli-related proteins was widespread among the genomes, although its function is unclear.

**IMPORTANCE** SAR11 clade members are among the most abundant bacteria on Earth. Their study is complicated by their great diversity and difficulties in being grown and manipulated in the laboratory. On the other hand, and due to their extraordinary abundance, metagenomic data sets provide enormous richness of information about these microbes. Given the major role played by phages in the lifestyle and evolution of prokaryotic cells, the contribution of several new bacteriophage genomes preying on this clade opens windows into the infection strategies and life cycle of its viruses. Such strategies could provide models of attack of large-genome phages preying on streamlined aquatic microbes.

## Abstract (precis)

SAR11 clade members are among the most abundant bacteria on Earth. Their study is complicated by their great diversity and difficulties in being grown and manipulated in the laboratory. On the other hand, and due to their extraordinary abundance, metagenomic data sets provide enormous richness of information about these microbes. Given the major role played by phages in the lifestyle and evolution of prokaryotic cells, the contribution of several new bacteriophage genomes preying on this clade opens windows into the infection strategies and life cycle of its viruses. Such strategies could provide models of attack of large-genome phages preying on streamlined aquatic microbes.

## INTRODUCTION

In marine ecosystems, bacteriophages (viruses that infect bacterial cells) are extremely abundant, with an estimated >10^10^ viral particles per liter of seawater (1, 2). Their lytic lifestyle is responsible for the mortality of nearly 10% to 50% of the microbial population per day (3). Therefore, it should not come as a surprise that bacteriophages are important players in the functioning of the marine microbial ecosystem. For example, they affect nutrient cycling through the “viral shunt” (4), influence microbial community composition and diversity (5), and drive host evolution, both by favoring genetic exchange and by predation pressure. The latter is of special importance as it favors high diversity at the population level, especially at loci that code for phage resistance traits (6, 7).

The SAR11 clade (including the order *Pelagibacterales*) is one of the most abundant bacteria in marine ecosystems, constituting approximately 20% to 40% of all planktonic cells in the oceanic photic zone (8). A particular subclade within SAR11 (LD12) is also important in freshwaters, lakes, and rivers, although less prevalent (9). Recently, a representative of this freshwater subgroup was isolated in pure culture and named *Candidatus* Fonsibacter ubiquis (9). Considering the facts described above, we would expect that members of this clade are prime targets for phage predation. To date, only 15 SAR11 phages have been isolated, all belonging to the order *Caudovirales* (10, 11). This order of viruses is the most prevalent in aquatic environments and can be divided into the families *Myoviridae*, *Siphoviridae*, and *Podoviridae* on the basis of their morphological characteristics (12). SAR11 phages belonging to the *Podoviridae* family are found more often both in pure culture (10, 11) and metagenomic collections (13–15) compared to the other two families. Most of these phages belong to the subfamily *Autographivirinae*, and it has been suggested that many are temperate phages that use tRNA genes as integration sites (11). Only one of the isolated SAR11 phages belongs to the *Myoviridae* family, and despite the abundance of cultivation‐independent metagenomic sequencing techniques, only four more myophage genomes have been found in the form of metagenome assembled viral genomes (MAVGs) (14). This scarcity of pelagimyophage (PMP) genomes is surprising, since several metagenomic studies from aquatic environments have shown that T4-like phages constitute the dominant fraction of the viral community (16–19).

The PMP genomes discovered thus far are all part of the *Tevenvirinae* subfamily. This subfamily of double-stranded DNA, contractile-tailed phages owe their name to their remarkable gene homology and genomic synteny to the well-studied Escherichia coli-infecting T-even phages, which are represented by T4 (20). Members of this subfamily have been isolated from a variety of hosts (21–24) and can be clustered into three phylogenetic groups based on the genetic divergence of the major capsid protein: Far T4, Near T4, and Cyano T4 (25). PMP HTVC008M is included within the Cyano T4 group (10), together with viral isolates of Sinorhizobium meliloti (23), Stenotrophomonas maltophilia (26), and the marine cyanobacteria *Synechococcus* and *Prochlorococcus* spp. (24). The latter group is known as the cyanomyophages (CMPs) and is the clade most closely related to HTVC008M. CMPs are generalist phages, successfully infecting hosts from different cyanobacterial species (27), and even genera (28). All CMPs share a set of core genes related to virion structure, DNA replication, and auxiliary metabolic genes (AMGs) (24, 29, 30), which are involved in supplementing host metabolism during infection (31).

Given their large genomes and complex morphology, myoviruses can provide rich information about their hosts and life cycle. In this study, we analyzed 26 new sequences of myophages that putatively infect the SAR11 clade retrieved by mining aquatic metagenomes. This alternative approach to culture-dependent methods has succeeded in discovering new viruses from uncultured microbes earlier (32, 33). Together, these findings increased sixfold the SAR11 myophage repertoire and allowed us to discover different PMP clades, including the first myophage specific of the freshwater genus *Ca*. Fonsibacter and the bathypelagic SAR11 phylogroup Ic (9, 34). This recovery effort has increased their genome diversity enough to be able to perform genomic comparisons with the closest well-studied CMPs to elucidate peculiarities of the PMP infection model.

## RESULTS

Figure S1A in the supplemental material shows the workflow that we used to recover sequences of myophages that putatively infect the SAR11 clade from several cellular metagenomic and viromic samples (see Table S1 in the supplemental material). In the end, we were able to recover 26 new PMP MAVGs that, together with the reference sequences, add up to 31 genomes (Table 1). Interestingly, 25 of the 26 new sequences have been recovered from the cellular fraction and not from the viral fraction, which could explain their poor representation in databases.

**TABLE 1:** Genomic features for the pelagimyophages analyzed in this study

PMP	Group	Mean igs (bp)^*a*^	Length (bp)	GC content (%)	No. of tRNAs	No. of genes	Complete- ness^*b*^	No. of matches^*c*^ to:		Habitat^*d*^	Sample type^*e*^	Reference(s)
								SAR11	PMP core			
HTVC008M	A	23.87	147,284	33.45	0	199	Yes (Cu)	9	23	M	C	10
Io7-C40	A	21.35	103,430	33.11	2	117	No	11	17	M	MG	13
MAVG02	A	25.5	157,661	33.98	0	216	Yes (Al)	10	20	M	MG	14
MAVG05	A	21.49	164,624	32.74	2	228	Yes (Al)	15	37	M	MG	14
PMP-MAVG-4	A	21.59	179,730	32.04	0	242	Yes (Al)	21	24	M	MG	93
PMP-MAVG-12	A	15.54	104,791	33.36	0	131	No	5	20	M	MG	92
PMP-MAVG-18	A	23.35	153,977	32.58	1	197	No	17	25	M	MG	93
PMP-MAVG-21	A	24.53	135,163	31.59	0	195	No	11	24	M	MG	93
PMP-MAVG-25	A	25.56	142,712	31.7	0	204	Yes (Al)	19	24	M	MG	93
PMP-MAVG-8	A	14.28	118,694	31.91	0	159	No	13	14	M	MG	91
PMP-MAVG-2	B	15.66	139,426	32.4	0	189	No	7	28	M	MG	92
PMP-MAVG-3	B	16.98	147,773	32.66	0	200	Yes (Al)	8	23	M	MG	14, 92
PMP-MAVG-14	B	18.64	136,460	32.92	2	186	No	11	27	M	V	91
PMP-MAVG-16	B	28.57	132,453	32.99	3	179	Yes (TR)	5	25	M	MG	93
PMP-MAVG-19	B	24.69	149,077	34.83	2	199	Yes (TR)	9	18	M	MG	93
PMP-MAVG-26	B	25.6	142,788	32.48	0	193	No	7	29	M	MG	91
PMP-MAVG-1	C	26.18	118,124	33.71	1	154	No	4	11	M	MG	41
MAVG04	C	26.64	159,588	34.12	2	211	Yes (Al)	5	12	M	MG	14
PMP-MAVG-9	C	21.81	124,621	33.95	1	165	No	6	10	M	MG	41
PMP-MAVG-10	C	13	127,706	32.6	0	177	No	8	15	M	V	91
PMP-MAVG-17	C	21.52	149,073	34.51	3	200	No	5	13	M	MG	93
PMP-MAVG-22	C	15.6	103,989	34.17	0	129	No	2	10	M	MG	93
PMP-MAVG-24	C	21.72	116,502	34.74	1	162	No	1	11	M	MG	93
PMP-MAVG-15	D	21.52	144,833	31.3	3	193	Yes (TR)	6	6	F	V	93
PMP-MAVG-20	D	21.3	122,912	31.08	3	174	No	8	6	F	V	93
PMP-MAVG-5	E	26.22	149,934	33.6	3	190	Yes (TR)	4	10	M	MG	41
PMP-MAVG-6	E	27.22	135,833	33.58	1	176	No	4	17	M	MG	41
PMP-MAVG-7	E	32.87	135,598	33.82	2	171	No	2	14	M	MG	41
PMP-MAVG-11	E	27.05	141,312	34.54	1	177	Yes (Al)	5	16	M	MG	41
PMP-MAVG-13	E	24.74	155,847	34.2	0	208	Yes (Al)	3	16	M	V	91
PMP-MAVG-23	E	19.87	110,977	34.96	2	146	No	4	10	M	MG	93

a Igs, intergenic spacer.

b How completeness was found is shown is parentheses: Cu, cultivated; Al, alignment; TR, terminal repeats.

c Protein matches, based on BLASTN hits with at least 70% similarity and an alignment length between 70% and 130% of the length of the smaller protein.

d M, marine; F, freshwater.

e C, culture; MG, metagenome; V, virome.

10.1128/mSystems.00905-19.1 FIG S1 Genome mining pipeline. (A) Workflow describing the steps used in the genome mining process, shown colored based on the step. (B) Contigs remaining in the analysis after each step, long with the average contig size. Each step includes a key to the workflow in panel A. Download FIG S1, PDF file, 0.2 MB. Copyright © 2020 Zaragoza-Solas et al. 2020 Zaragoza-Solas et al. This content is distributed under the terms of the Creative Commons Attribution 4.0 International license.

10.1128/mSystems.00905-19.6 TABLE S1 Metagenomic database samples utilized in this study. Download Table S1, PDF file, 0.4 MB. Copyright © 2020 Zaragoza-Solas et al. 2020 Zaragoza-Solas et al. This content is distributed under the terms of the Creative Commons Attribution 4.0 International license.

### Genomic features.

**FIG 1:**
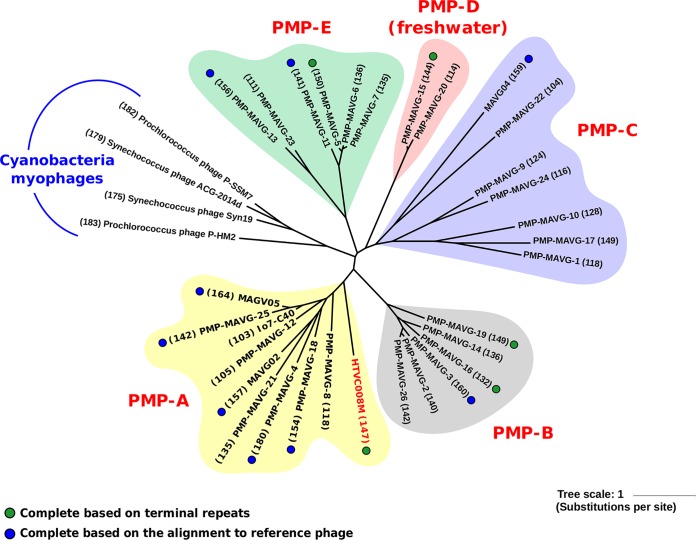
Unrooted phylogenomic tree of concatenated conserved proteins (terminase small subunit, terminase large subunit, tail tube monomer, tail tube monomer, baseplate wedge protein gp8, and VrlC protein) found in pelagimyophages (PMPs) and in the cyanomyophage outgroup. The reference cultured PMP is highlighted in red. The size (in kilobases) of each MAVG is shown in parentheses next to each branch, with complete PMP MAVGs marked with solid circles.

MAVG completeness was verified either by the presence of identical repeated sequences (>10 nucleotides [nt]) at the 5′- and 3′-terminal regions or by showing a similar synteny and gene content to the cultivated PMP HTVC008M (10). The genome size of the 13 complete genomes ranges from 132 to 164 kb (Table 1). To study the relationships of the recovered phages, the 31 PMP genomes were compared in a phylogenomic tree using four CMP genomes as an outgroup. The five proteins common to all 35 genomes (large and small subunits of terminase, VrlC protein, tail tube monomer gp18, and baseplate wedge protein gp8) were merged into a concatemer. The phylogenomic tree clustered PMPs into five different groups (PMP-A to PMP-E), with group PMP-A containing the reference phage HTVC008M (Fig. 1). Host assignment within different SAR11 subclades was not possible (except for group D [see below]) due to (i) lack of tRNA genes (only 18 genomes had them, and the ones present were all under 95% identity to SAR11 known tRNAs), which suggests that either we do not have genome representatives for the hosts they infect, or they have a broad host range, (ii) similarity of shared proteins provided inconclusive results (same identity to distantly related host-groups) and (iii) there is only one report of a CRISPR-cas system in SAR11, which is found only in the bathypelagic ecotype Ic (34). The enormous diversity of the SAR11 clade probably complicates the process of host assignment.

**FIG 2:**
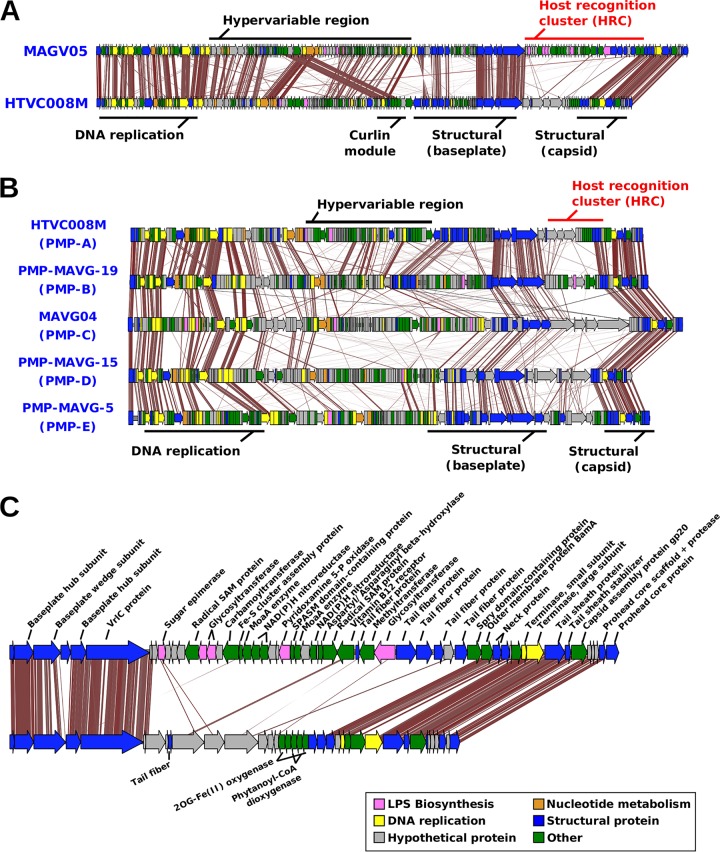
Alignment of pelagimyophage genomes (tblastx, 30% identity). (A) Whole-genome alignment of two PMP-A group genomes. The different modules and hypervariable regions are labeled with black lines over the genomes, while the host recognition module (HRC) is highlighted in red. (B) Whole-genome alignment of a complete representative of each PMP group. (C) Close-up view of the HRC. Genes are colored according to their predicted function.

**FIG 3:**
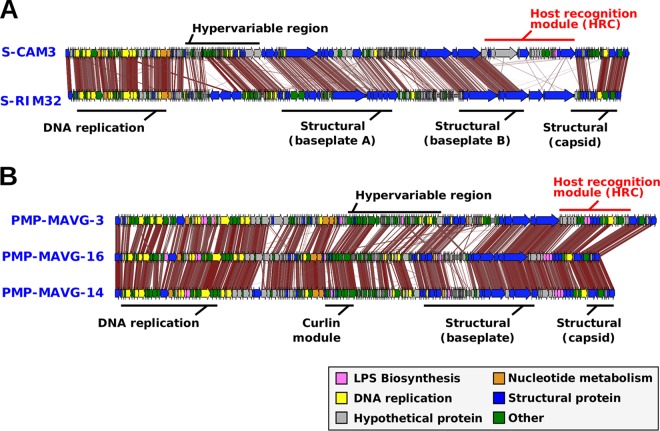
Alignment of pelagimyophage and cyanomyophage (CMP) phages (tblastx, 30% identity). Gene modules are labeled with black lines over the genomes, with the host recognition cluster highlighted in red. (A) Alignment of two CMPs. (C) Alignment of three PMPs from group PMP-B with a similar HRC.

Figure 2A shows the alignment of two genomes of group PMP-A (one of them the pure culture HTVC008M), while alignments of one representative genome from each cluster are shown in Fig. 2B. Overall, synteny was well preserved in all sequences once they were rearranged to start from the major capsid gene (*gp23*), and all of the sequences displayed the characteristic patchwork architecture of the *Tevenvirinae* subfamily, with remarkably conserved core modules (DNA replication and virion structure) separated by variable regions, designated as hypervariable (21, 35) (Fig. 2A and B). The most remarkable feature is the presence of a large nonsyntenic island located in the middle of the structural region, always between the VrlC gene and the neck protein gene *gp14* (Fig. 2C). On the basis of its variable character and the presence of tail fibers, we have designated this variable region the host recognition cluster (HRC) (Fig. 2C). In other T4-like phages, this region contains only the tail fiber module (30, 35). This large hypervariable region has been already described in CMPs, usually containing several structural genes and AMGs (30). In PMPs, this region is larger (mean HRC size of 44.6 kb versus 34.2 kb in CMPs), and contains, along with the expected tail fiber genes, a large number of genes seemingly unrelated to the tail fiber module, the most conspicuous of which are several glycosyltransferases, typically involved in the synthesis of the O chain of the lipopolysaccharide that is located in the outer layer of the Gram-negative cell envelope (24, 36) (Fig. 2C). In PMPs, 63 out of the 162 lipopolysaccharide (LPS)-related proteins found are inside the HRC, while CMP HRCs have more identifiable tail fiber-related proteins. However, the latter could be attributed to the fact that CMPs are better represented in the sequence databases and are thus easier to annotate. The comparison of the CMP and PMP genomes showed strong conservation of all modules, including the HRC (Fig. 3A). However, unlike the latter, in some CMP genomes, the baseplate module is divided by another plastic region (Fig. 3A).

The two most similar complete genomes were MAGV3 and MAGV16, found in cluster B (average nucleotide identity [ANI] of 72.0% and coverage of 38.6%), although they were assembled from the Western Arctic ocean and the Mediterranean Sea, respectively (Fig. 3B). In the case of these two, the HRC was much more similar and differed only by the addition of some gene cassettes related to radical SAM (*S*-adenosyl-l-methionine) proteins (Fig. 3B). Their comparison seems to indicate that the divergence of this region is a gradual process rather than a complete replacement, as described for replacement flexible genomic islands in prokaryotic cells (37). The genes located downstream from VrlC, which are the tail fibers in most genomes, show high similarity, indicating a possible host overlap of these two phages.

### Recruitment from cellular metagenomes and viromes.

**FIG 4:**
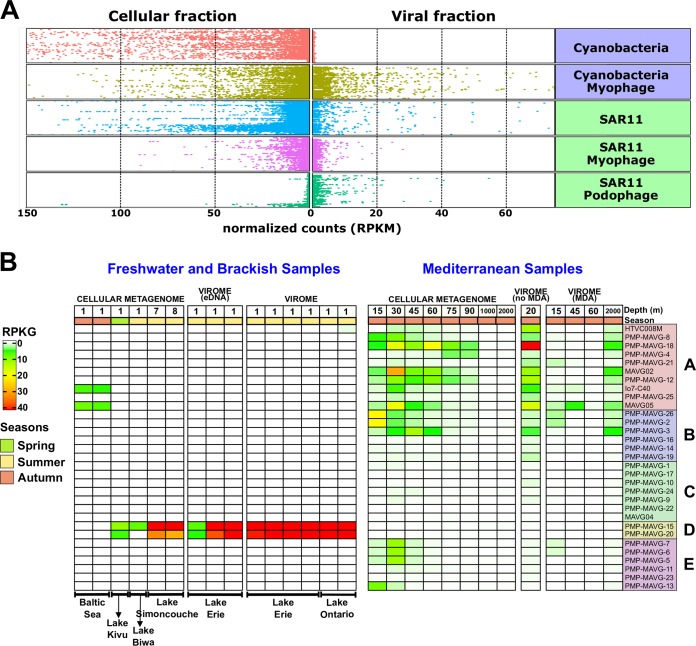
Recruitment of pelagimyophages. (A) Relative abundance of PMPs reads in Mediterranean, Geotraces, and *Tara* Oceans metagenomes and viromes is shown along with the abundances of SAR11 bacteria, SAR11 podoviruses, and *Cyanobacteria* and their myophages. The horizontal axis shows the normalized count of reads per kilobase pair of genome and megabase pair of metagenome (RPKM), while the vertical axis shows the sampling stations (like in reference 33). (B) Heatmap of abundance of PMPs in freshwater and Mediterranean cellular metagenomes and viromes. Normalization of the abundance was performed by calculating RPKG (reads recruited per kilobase of the genome per gigabase of the metagenome).

To evaluate the abundance and elucidate possible patterns of distribution of these phages, we performed recruitment analysis by comparing each sequence to 395 metagenomes from Mediterranean depth profile (38, 39), *Tara* Oceans (40) and Geotraces (41) data sets as well as several freshwater metagenomes (see Materials and Methods). We considered only those samples where at least one PMP recruited more than five reads per kilobase of genome and gigabase of metagenome (RPKG) with an identity of >95%. PMP genomes showed a wide, if uneven, oceanic distribution along the *Tara* Oceans transect (40) (Table S2). All genomes except the freshwater PMP-D group (see below) recruited significantly in several marine samples from different geographic regions, with maximum recruitment typically found in the 5-to-45-m-depth range. Figure 4A shows the recruitment of both families of SAR11 phages (*Podoviridae* and *Myoviridae*) and their host in both the cellular and viral fractions from *Tara* Oceans. In addition, we have also included the other most relevant and widespread marine group, *Cyanobacteria*, and their myophages. While the presence of podophages was mainly restricted to viromes, both groups of myophages were present in both fractions (cellular and viral) (Fig. 4A), although pelagimyophage genomes recruited significantly more from cellular metagenomes than from viromes. The abundance of viral DNA in the cellular fraction indicates that a high number of microbial cells are undergoing the lytic cycle, which acts as a natural amplification of viral DNA (13, 14). Another interesting observation was that a significant amount of SAR11 DNA was present in viromes, probably because some SAR11 cells might be small enough to pass through the 0.2-μm filter used frequently to retain bacteria (Fig. 4A) (8, 42). A latitude transect from 50°N to 50°S in the West Atlantic Ocean was analyzed using the Geotraces database (41). However, latitude did not seem to be a significant factor in their distribution (Table S3).

10.1128/mSystems.00905-19.7 TABLE S2 Pelagimyophage recruitment values for *Tara* Oceans metagenomes and viromes. Normalization of the abundance was performed by calculating RPKG (reads recruited per kilobase of the genome per gigabase of the metagenome). Download Table S2, XLSX file, 0.04 MB. Copyright © 2020 Zaragoza-Solas et al. 2020 Zaragoza-Solas et al. This content is distributed under the terms of the Creative Commons Attribution 4.0 International license.

10.1128/mSystems.00905-19.8 TABLE S3 Pelagimyophage recruitment values for Geotraces metagenomes. Normalization of the abundance was performed by calculating RPKG (reads recruited per kilobase of the genome per gigabase of the metagenome). Download Table S3, XLSX file, 0.1 MB. Copyright © 2020 Zaragoza-Solas et al. 2020 Zaragoza-Solas et al. This content is distributed under the terms of the Creative Commons Attribution 4.0 International license.

The recruitment results as a whole suggest that PMP amplification is biased, as this group of genomes always recruited much more from cellular metagenomes than from viromes. The nature of this bias (either biological or technical) is still unclear. We also observed significant differences in recruitment values between the Mediterranean viromes treated with multiple displacement amplification (MDA) and those that had not been amplified (Fig. 4B). Although there is no direct evidence of their effect over myoviruses, MDA amplification might have played a part in these differential recruitment. MDA has been reported to be biased toward certain nucleic acid structures and sequences (43, 44).

However, we were able to distinguish some groups with different patterns of recruitment. One genome of group PMP-A (PMP-MAVG-4) predominantly recruits below 200 m in both the Geotraces and TARA data sets, supporting its association to bathypelagic *Pelagibacterales* clade Ic (34) (Fig. S2 and Tables S2 and S3), although the assignment is tentative, since it could not be proven by sequence analysis. Due to the scarcity of samples from the deep ocean, we can confirm its presence only in temperate zones of the Pacific and Atlantic Oceans (Tables S2 and S3). In Mediterranean samples, it appears only in areas below the deep chlorophyll maximum (75 to 90 m) but not at bathypelagic depths, probably due to the Mediterranean relatively warm water column, although Ic representatives have been detected there (Fig. 4B) (45). Unique genes to this putatively “deep ecotype” include a GMP reductase and various genes involved in heme biosynthesis (coprophyrinogen oxidase, porphobilinogen deaminase) as well as a formate dehydrogenase, an enzyme that transforms formate into CO_2_ and 2H^+^ (46). This could be an adaptation to generate a proton gradient in the absence of light, as SAR11 cells can generate it via rhodopsins. Two other PMP-A representatives, MAGV05 and Io7-C40, showed tolerance for brackish waters, as demonstrated by their recruitment from Baltic Sea cellular metagenomes (Fig. 4B). Group D recruits only from freshwater samples, making them the first described freshwater myophages of the SAR11 clade (see below) (Fig. 4B). Linear recruitments (Fig. S3A) showed that although genomes recruit along their entire lengths, most of the reads were recruited at more than 99% identity. The genome regions that recruit vertically down to 80% identity correspond to the structural and DNA replication-related genome regions described previously, which are very well conserved among all the members of the subfamily (24, 35). The HRC usually underrecruited, indicating the highly variable nature of this region (Fig. S3A). The same pattern was observed in cellular metagenomes and viromes with and without MDA (Fig. S3A).

10.1128/mSystems.00905-19.2 FIG S2 Heatmap of abundance of pelagimyophages in GEOTRACES Cruise GA3 samples. Download FIG S2, PDF file, 0.2 MB. Copyright © 2020 Zaragoza-Solas et al. 2020 Zaragoza-Solas et al. This content is distributed under the terms of the Creative Commons Attribution 4.0 International license.

10.1128/mSystems.00905-19.3 FIG S3 (A) Linear recruitments of various pelagimyophages against various metagenomes and viromes. The host recognition cluster (HRC) is indicated by a red rectangle. (B) Isoelectric point versus density plot of marine (HTVC008M) and freshwater (PMP-MAVG-15) pelagimyophages. (C) Genome alignment of HTVC008M and PMP-MAVG-15. Genomic modules are indicated by black bars over the genomes, with the HRC highlighted in red. Download FIG S3, PDF file, 2.5 MB. Copyright © 2020 Zaragoza-Solas et al. 2020 Zaragoza-Solas et al. This content is distributed under the terms of the Creative Commons Attribution 4.0 International license.

### First genomes of PMPs infecting *Ca.* Fonsibacter.

Genomic analysis of the two genomes in group PMP-D showed that both contained tRNA genes with the best match to tRNAs from the recently isolated *Candidatus* Fonsibacter ubiquis LSUCC0530, a member of the LD12 subclade (9). Metagenomic recruitment showed clear evidence that group PMP-D was associated with freshwater samples (Fig. 4B). To our knowledge, these are the first genomes of myophages that putatively infect *Ca*. Fonsibacter (fonsimyophages). Both are remarkably similar to each other but present different degrees of completeness. PMP-MAVG-15 is considered complete, while PMP-MAVG-20 is lacking the DNA replication module. Recently, a shift toward basic values was described in the relative frequency of predicted isoelectric points when comparing freshwater and marine microbes (47). Along these lines, we found a significant difference in PMPs infecting *Ca*. Fonsibacter compared to the reference genome HTVC008M (Fig. S3B). However, synteny was well preserved between marine and freshwater groups (Fig. S3C).

10.1128/mSystems.00905-19.4 FIG S4 (A) Cooccurrence networks of cyanomyophage and pelagimyophage genomes. Each node represents a protein cluster (PC), while each edge indicates that the two nodes it connects are present in the same operon in at least a pair of genomes. Edge thickness indicates the number of genomes the two PCs are present in the same operon. Each node is colored according to its predicted function. (B) Phylogenetic trees of two curli operon proteins (CsgF and CsgG) from pelagimyophages, *Alphaproteobacteria*, and *Gammaproteobacteria*. Pelagimyophage genes are highlighted in red. Download FIG S4, PDF file, 0.2 MB. Copyright © 2020 Zaragoza-Solas et al. 2020 Zaragoza-Solas et al. This content is distributed under the terms of the Creative Commons Attribution 4.0 International license.

Recruitments show the recovered fonsimyophages to be present in various lakes from Canada (Erie, Ontario, Simoncouche) in both the cellular and viral fraction (Fig. 4B). We also found recruitment matches at lower identity (<80%) in other freshwater samples (Lake Biwa, Lake Kivu). Linear recruitments for group D phages against freshwater viromes are different from those originating from their marine counterparts (Fig. S3), showing that diversity in fonsimyophages is lower than that of the marine PMPs. This fact might reflect the reduced intrapopulation diversity of their host compared to other SAR11 subclades (9).

Gene content comparisons between marine or freshwater SAR11 PMPs shed little light on possible adaptations to the latter. However, the freshwater genomes do not contain genes related to LPS, substrate transport, radical SAM proteins, or the curli operon (see below). Nevertheless, it has some unique genes, such as *speH* (involved in polyamine salvaging), various genes involved in lipid biosynthesis (*fabF*, stearoyl-coenzyme A [CoA] desaturase) and a 2OGFeDO superfamily protein, which catalyzes nucleic acid modifications (48, 49). Strikingly, some proteins core to all PMPs (peptide deformylase, ribosomal protein S21, and aspartyl/asparaginyl beta-hydroxylase) are present in group PMP-D but are different enough to be separated in independent protein clusters.

### Comparative genomics.

To maximize our ability to annotate phage proteins, we clustered orthologous genes into protein clusters (PCs) and annotated their function following a consensus-based approach (see Materials and methods). The PCs with the most differences in abundance between PMPs and CMPs have been collected in Table S4. Furthermore, to examine the organization of the PCs into operons in both groups of phages, we built a cooccurrence matrix (Fig. S4A), which links genes if they are in the same operon. Previously described methods to detect middle and late promoters in CMPs (24) did not provide satisfactory results when applied to PMPs, so we delimited operons by terminators and strand changes (see Materials and Methods). The cooccurrence matrix reveals differences in the structural organization of the operons containing conserved PCs. While structural operons contain only structural or hypothetical proteins, operons containing DNA metabolism genes are more diverse, containing AMGs of various types. Furthermore, genes involved in the same function are not in the same operon unless they are subunits of the same protein or the presence of one is meaningless without the other. An example of this phenomenon would be the photosynthesis AMGs in CMPs. Photosystem II D1 and D2 subunits are always in the same cluster, but the reaction center protein PsbN is not.

10.1128/mSystems.00905-19.5 FIG S5 Phylogenetic terminase tree. The branch with the reference terminase genes is shown in red. Download FIG S5, PDF file, 0.1 MB. Copyright © 2020 Zaragoza-Solas et al. 2020 Zaragoza-Solas et al. This content is distributed under the terms of the Creative Commons Attribution 4.0 International license.

10.1128/mSystems.00905-19.9 TABLE S4 Protein clusters (PCs) with size of >10 genes and a log fold difference of at least 1. Normalized gene counts were calculated as follows: (total number of genes in all genomes of the group/total genomes in the group). Download Table S4, PDF file, 0.2 MB. Copyright © 2020 Zaragoza-Solas et al. 2020 Zaragoza-Solas et al. This content is distributed under the terms of the Creative Commons Attribution 4.0 International license.

**(i) Structural genes.** Structural modules are well conserved among both groups of phages, as we identified homologs for the majority of typically conserved structural capsid and tail proteins. Despite the structural conservation of core components in all *Tevenvirinae* phages, we were unable to identify some conserved but highly divergent proteins, like the tape measure or tail fiber proteins. The structural region with the most differences compared to the T4 phage was the baseplate. Both groups contain homologs for a large number of the genes involved in the internal structure of the baseplate of T4-type phages (50), which is involved in baseplate assembly, initiation, and sheath contraction (51). A remarkable difference is the absence of T4 Gp7, which appears to be substituted in both groups of phages by the VrlC protein. VrlC is particularly meaningful, as it is considered an integral component of the two-layered baseplate structure (52, 53), so we can predict that both groups possess this type of baseplate. The other regions of the baseplate appear to be less conserved. Within this large structural operon, we also found various unidentified structural proteins that contain domains linked to carbohydrate-binding and host recognition (specifically, YHYH domains, concanavalin A domains, triple collagen repeats, major tropism determinant domains, and YadA domains) (54–58). These putative receptor-binding proteins could be part of the tail fiber complex or the baseplate, as double-layered baseplates have been reported to contain these kind of proteins (52). Last, the *gp5* gene shows a much larger divergence than the VrlC protein, with both groups of phages coding for various gp5 PCs. As gp5 is involved in cell puncturing and local cell wall degradation (59), we can assume that the differences in gp5 PCs are an adaptation to the specific cell wall of the host.

**(ii) DNA transcription and translation.** Transcription regulation in PMPs seems to be quite similar to that of CMPs, with both groups lacking homologs to the T4 genes involved in regulating early and middle transcription (*alt*, *modA*, *modB*, *asi*, and *motA*) (60, 61). Some genomes of group PMP-A code for an homolog of the L12 ribosomal protein, which is the binding site for several factors involved in protein synthesis (62), and a tRNA(Ile)-lysidine synthetase, which is an uncommon nucleoside usually seen only in tRNA and involved in solving differences between the elongation methionine tRNA and isoleucine tRNA (63). The most significant difference between both groups of phages related to the translation process is that the latter group codes for a homolog of the 30S ribosomal protein S21. This protein is responsible for the recognition of complex mRNA templates during translation and has been described only as an AMG in HTVC008M (64, 65). S21 is not part of any specific gene cluster, which, assuming the protein follows the same rules as the other AMGs, suggests that no other viral factors are required for its functionality.

### Auxiliary metabolic genes.

CMPs frequently contain AMGs, homologs of host genes, to modify host metabolism during infection (66). We have analyzed the occurrence of this type of genes in the PMP genomes and compared it with the occurrence in CMPs (Table S5), which have been widely studied (67).

10.1128/mSystems.00905-19.10 TABLE S5 AMGs detected in the analyzed genomes. Download Table S5, PDF file, 0.2 MB. Copyright © 2020 Zaragoza-Solas et al. 2020 Zaragoza-Solas et al. This content is distributed under the terms of the Creative Commons Attribution 4.0 International license.

Both groups of phages had the three classic AMGs involved in nucleotide biosynthesis (*cobS*, *cobT*, both subunits of ribonucleotide reductase) (66, 68) (Table S5). However, Both PMP-A and PMP-B groups code for the adenylate kinase *adk*, which is involved in the interconversion between adenine nucleotides (69), while group C has two different thymidylate synthases and a deoxycytidylate CMP deaminase, which provides the substrate for both (70, 71) (Table S4). A peptide deformylase involved in protein maturation was present in all PMPs in the core genome, inside a DNA metabolism operon, while in their cyanobacterial counterparts, it was found only in a few and inside the flexible genome, together with the photosystem AMGs (72).

We found fewer genes dedicated to regulation in PMPs than in CMPs. Typical CMP regulation AMGs such as *mazG* are absent in PMPs, and regulation genes shared by both groups such as the Pho regulon *PhoH* or Sm/Lsm RNA-binding proteins are more abundant in CMPs than in PMPs (Table S5). However, genes related to the *sprT* family (a gene involved in the regulation of the stress factor BolA) are much more prevalent in PMPs than in CMPs. *bolA* has many effects on cell morphology, cell growth, cell division, and biofilm development in the stationary phase and under starvation conditions (73). These differences in regulatory proteins are not surprising, since it has been proposed that SAR11 cells are not as tightly regulated as cyanobacteria (8); hence, their regulatory systems would be significantly different (as mentioned above, the starvation system *mazE*/*mazG* does not exist in SAR11 but it is present in picocyanobacteria) (8). Regulation in SAR11 seems to be less dependent on proteins, being directed by riboswitches and other small mRNA (smRNA) molecules instead (8). However, a search of these regulatory mRNAs with the tool Riboswitch Scanner (74) found no evidence of their presence in neither group of phages.

Another type of AMG found in PMP genomes are genes related to the production of the O-chain of bacterial lipopolysaccharides, usually found as part of the HRC, but also distributed along the genome in clusters of two or three genes. This category of genes is also found in CMPs but is much less abundant. The LPS-related genes are either enzymes involved in the synthesis of deoxy-sugars to use as building blocks (*rfaE*, UDP-glucose 6-dehydrogenase) (75, 76) or are glycosyltransferases, involved in adding specific sugar residues to a molecule (77). Glycosyltransferases in bacteriophages are involved in the glycosylation of viral DNA to protect against the host restriction-modification systems or in the modification of the O-antigen chain of the host to protect against coinfection by other phages (36). Considering that the glycosyltransferase family most represented in PMPs is GT8, which is mainly involved in LPS biosynthesis (78), and that only one SAR11 genome out of more than 100 sequenced thus far codes for a restriction-modification system (79), it seems likely that glycosyltransferases in this group are involved in the modification of the O-chain of their host.

### Curli operon.

Between the DNA replication and structural modules, there is a hypervariable region containing a variable number of genes with little synteny among the different PMP representatives (Fig. 2A and Fig. S2A). Within this variable region, we found two homologs of the type VIII secretion system (TSS VIII) present in all PMP groups but the fonsimyophages (Fig. 2). To our knowledge, this is the first report of phages that code for proteins of this secretion system. The cooccurrence network shows that these proteins are part of a well-defined operon that includes the proteins CsgF, CsgG, two hypothetical proteins and a curli-associated protein. The phylogenetic tree of the PMP and bacterial curli proteins clustered them closer to the *Alphaproteobacteria* representatives (Fig. S4B ).

TSS VIII has not been detected in SAR11, but it has been described in other bacterial groups (80) as the transporter of curli, surface-associated amyloid fibers mainly involved in adhesion to surfaces, biofilm formation, and interaction with host factors and the host immune system (81, 82). The two proteins identified as part of the TSS VIII in PMPs are CsgF, an extracellular chaperone involved in anchoring curli fibers to the outer membrane (83), and CsgG, which form the outer membrane diffusion channel (84). Both hypothetical proteins in the operon are of the same size, similarly to *csgA* and *csgB* genes (85), while the curli-associated protein is of the same size as CsgE, although no similarity could be detected at the sequence level or predicted structural level. Several experiments have shown that the only proteins required for curli phenotype expression are CsgA, CsgB, CsgF, and CsgG (CsgE increases almost 20-fold the amount of curli released, but it is not essential) (83, 86). Therefore, CsgA and CsgB are the only proteins missing in PMPs for the infected cells to express a curli phenotype.

## DISCUSSION

The kind of bioinformatic approach utilized here can be applied to other microbes difficult to cultivate but with some isolates already sequenced. The diversity of sequences retrieved indicate that similar methods could provide much more complete pictures of the biodiversity of viruses infecting relevant but hard to grow microbes such as SAR11. In this case, its prevalence in superficial waters of the ocean and other aquatic habitats played in our favor, and we have been able to uncover a remarkable diversity of viral entities different from the cultivated reference. It seems clear that the amplification of PMPs in viromes is negatively affected by one or more biases, with MDA amplification being a prime suspect, and the same might be true for other myoviruses. This application of metagenomics complements culture to capture more phage diversity in natural environments (14).

The host cells belonging to the SAR11 clade are characterized by marked streamlining of the genomes (8). Myophages, on the other hand, are very large phages with big and complex genomes. In fact, the ones described here are even more complex than E. coli phage T4, with a large host recognition hypervariable island and novel sets of AMGs. They are actually closest to CMPs, a group of myophages whose host range also includes streamlined microbes (e.g., *Prochlorococcus*) inhabiting a similar habitat, an interesting convergence considering the phylogenetic distance between the hosts. Among the special features of the PMP genomes, it is remarkable that the large hypervariable region involved in host recognition in addition to several tail fibers, often contained glycosyltransferases, which might be involved in surface alterations that could lead to changes in phage recognition, preventing coinfection by other phages preying on the same host. That these large phages of SAR11 require a change in the host surface is not surprising, given the potentially sharp competition with, for example, SAR11 podophages that have much larger burst sizes (42 ± 7 versus 9 ± 2 for the cultured representatives) (10, 11). The genes provided by the phage might induce a change in the structures responsible for phage recognition and act as a serotype conversion mechanism to avoid superinfection by other phages (87). Similar mechanisms have been described for other marine and nonmarine podoviruses (88–90).

PMPs are, to our knowledge, the first phages that code for a partial curli-secreting system. The origin of this operon is unclear, since so far, the TSS VIII secretion system has not been described in the SAR11 clade. However, its remarkable similarity to the TSS VIII operon described in *Alpha*- and *Gammaproteobacteria* suggests that it is a product of a lateral transfer event. The function of such a system in viruses is also a mystery. The only two proteins identified as part of the TSS VIII in PMPs are CsgF and CsgG, which implies that if no other proteins in the operon are functional, it would code for only an extracellular chaperone and a pore-forming complex, respectively. The CsgG pore is too small to allow for virion exit (the CsgG pore has 40-Å inner diameter, while the HTVC008M capsid diameter is 550 Å) (10, 86), and the only report of functional amyloids in viruses was in eukaryotic viruses, where they have the role of inhibiting programmed cell death of their eukaryotic host by sequestering effector proteins (89), which does not require the presence of the curli transporter. The simplest explanation would be that the pore structure might enhance the uptake of larger molecules. However, this does not explain the presence of CsgF, as it is not needed for the assembly of CsgG (82, 86) or the other genes present within the operon. Another, bolder hypothesis would be the involvement of these genes in the production of myeloid-like fibers. Some of the hypothetical proteins in the curlin cluster could be functional equivalents of CsgA and CsgB (86). If this were the case, they might induce aggregation, facilitating the acquisition of new host cells to the released virions. Thus, the curli gene cluster would act as a capture mechanism by retaining in close proximity the recently divided cells, that would be successive hosts, leading to a much larger phage offspring. This strategy could be called “sibling capture,” and would be highly desirable in diluted environments such as the pelagic habitat in oligotrophic waters.

## MATERIALS AND METHODS

### Genome mining strategy and output.

Following the workflow shown in Fig. S1A in the supplemental material, the reference cultivated PMP genome (HTVC008M) (10) and metagenomic PMP sequences MAVG-2, MAVG-4, MAVG-5, and Io7-C40 (14), were used as bait to comb through a vast quantity of contigs derived from several metagenomic and viromic samples (Table S1) (13, 14, 41, 91–94). First, a hidden Markov model (HMM) made from an alignment of *terL* gene sequences was used to identify viral contigs larger than 5 kb. The *terL* gene from the extracted contigs was then used to construct a phylogenetic tree (Fig. S7A). The position of the *terL* gene of the reference PMP in this tree was then used to recover a set of candidate contigs (Fig. S1B and S5). As mentioned previously, the closest group to PMPs are CMPs, which are expected to be present in significant quantities in the surveyed metagenomes. To remove all CMP-related contigs from the candidates, two collections of gene clusters were built, (i) one of them derived from 28 CMP genomes downloaded from the NCBI Refseq database (95) and (ii) another derived from the reference PMP genomes. Gene clusters shared between both collections were removed. HMMs built from both cluster collections were used to classify the contigs, keeping only those that had at least a match to a PMP gene cluster and no matches to any CMP gene cluster (Fig. S1).

### MAVG cross-assembly.

The contigs obtained from the genome-mining step were subjected to a cross-assembly step. Identical sequences were removed from the analysis, always keeping the longer contig if they did not have the same length. Contigs were then separated into bins of overlapping contigs based on an all-versus-all comparison (Fig. S1). Next, the bins were assembled manually into MAVGs as described previously (14) provided that (i) overlaps between contigs had a nucleotide sequence identity of >99%, an alignment length of >1,000 nt, and gaps of <10 nt, (ii) all overlaps were corroborated by more than two contigs, and (iii) sample metadata were ecologically coherent for all involved contigs (for example, not assembling contigs from freshwater and marine samples together). After this cross-assembly step, we obtained 14,748 sequences with an average length of 28 kb (Fig. S1B). Finally, contigs recovered were filtered by size (>100 kb), GC content (30 to 35%, which is the GC% range of the host), the number of proteins matching to SAR11 (>70% of identity), and tRNA gene matches (>95% of identity).

### Recruitment analysis.

To assess the distribution and abundance patterns of the recovered PMP MAVGs, the genomes were recruited using BLASTN (96) against the *Tara* Oceans metagenomes (40, 91), Geotraces cellular metagenomes (41), and the Mediterranean metagenomes described previously (14, 39). PMP group PMP-D were also recruited against the virome data sets they were recovered from (97) and against samples from other freshwater environments (Lake Biwa [98], Lake Simoncouche [99], Lake Kivu [GOLD Study identifier {ID} Gs0127566], Baltic Sea [100]). Normalization was performed by calculating RPKG (reads recruited per kilobase of the genome per gigabase of the metagenome) so recruitment values could be compared across samples. For linear metagenomic recruitments, metagenomic reads were aligned using BLASTN, with a cutoff of 70% nucleotide identity over a minimum alignment length of 50 nucleotides. The resulting alignments were plotted using the ggplot2 package in R. Figure 3A (cellular fraction versus viral fraction plot) was plotted following the scripts included in reference 33.

### Phylogenetic tree of the recovered genomes.

Common proteins to all 35 genomes were calculated using the GET_HOMOLOGUES (101) software package. The five common proteins identified were concatenated and aligned using MUSCLE (102) and a maximum-likelihood tree was then constructed using RAxML (103) with the following parameters: “-f a” algorithm, 100 bootstrap replicates, PROTGAMMAJTT model.

### Protein isoelectric point determination.

To determine the isoelectric point distribution patterns of the phage genomes, calculations of all predicted proteins for both genomes were calculated with the Pepstats software from the EMBOSS package (104). The resulting isoelectric point values were plotted using the ggplot2 package in R.

### Genomic pairwise comparison.

Average nucleotide identity (ANI) and coverage between a pair of genomes were calculated using the Jspecies software with default parameters (105).

### Genome annotation.

Genes and tRNAs were predicted using Prodigal (106) and tRNAscan-SE (107), respectively. Functional annotation of predicted genes followed a consensus-based approach. First, the genes from all PMPs and the reference CMPs were annotated against the uniref90 protein database (108) (using DIAMOND [109]) and the CDD (110) and pVOG (111) HMM databases (using hmmscan [112]). For each database, we assigned to each gene sequence the best hit with an E value of at least <10^−5^ and an alignment length of between 70% and 130% of the query length. Genes were then clustered using GET_HOMOLOGUES (101) and the annotations for each cluster were manually curated to ensure that the annotations were coherent for all genes in the cluster. In the cases where we found discrepancies, the second and third best hits were used to verify the annotation. Finally, the remaining clusters without annotation were compared against the PDB HMM database (113) using hhblits (114). Clusters with less than 10 sequences were first inflated by using the uniclust30 (115) database.

### Cooccurrence matrix.

Terminator sequences were predicted for both CMP and PMP genomes using Transterm_HP (116), while early promoter sequences were predicted using BPROM (117). Prediction of middle and late promoter sequences was attempted following the steps described previously (24) but was unsuccessful in PMP genomes. Genes that pertain to a protein cluster (obtained in the genome annotation step) in each genome were then grouped into operons based on terminator positions and strand changes. These operons were then used as the basis for a cooccurrence matrix. Two protein clusters (nodes) were linked to each other if they were present in two genomes and were part of the same operon, with edge strength representing the number of genome pairs where this was the case. Edges with edge strength representing 0.05% of the total were removed from the matrix. The matrix was then used to build a network in Cytoscape (118). The add-on ClusterMaker2 (119) was used to separate the cooccurrence network into clusters (MCL algorithm, 2.5 granularity).

### Data availability.

Viral sequences presented in this article have been submitted to NCBI and are available under BioProject accession number PRJNA588231.
