# Supplementary material for: Metagenome Mining Reveals Hidden Genomic Diversity of Pelagimyophages in Aquatic Environments
Source: mSystems. 2020 Feb 18;5(1):e00905-19. doi: 10.1128/mSystems.00905-19 (PMC7029224; doi:10.1128/mSystems.00905-19)
Supplement: FIG S1 [file mSystems.00905-19-sf001.pdf]

Genome mining flowchart

A

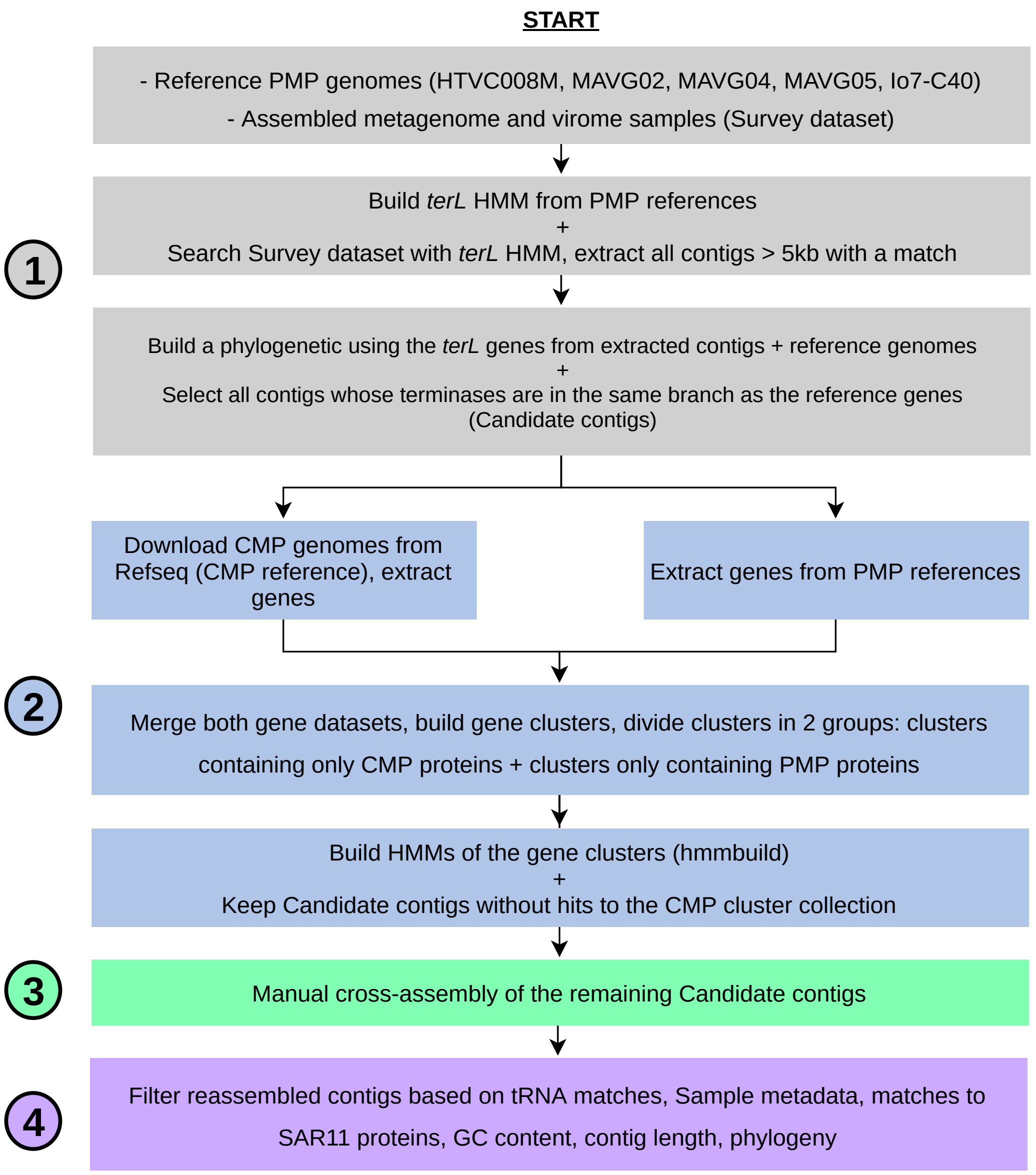

B

	Step	#Contigs	Average contig size (kbp)
1	*terL* hmm filtering	126,467	12.56
2	Gene cluster hmm filtering	30,331	25.89
3	Manual cross-assembly	28,431	26.42
	Duplicate removal	14,748	28.30
4	Contigs > 100kbp	688	152.05
	GC% between 30 – 35	197	143.03
	Phylogeny + Gene filtering	31	137.19
