# Supplementary material for: Metagenome Mining Reveals Hidden Genomic Diversity of Pelagimyophages in Aquatic Environments
Source: mSystems. 2020 Feb 18;5(1):e00905-19. doi: 10.1128/mSystems.00905-19 (PMC7029224; doi:10.1128/mSystems.00905-19)
Supplement: TABLE S1 [file mSystems.00905-19-st001.pdf]

Table S1. Metagenomic databases samples utilized in this study.

Name	Type	Habitat	Reference	Raw size (Gbp)1	Number of contigs > 5kb
GEOTRACES	Metagenome	Marine	[1]	7,152	703,045
HOT / BATS Time Series	Metagenome	Marine	[1]	2,206	225,141
Malaspina	Metagenome & Virome	Marine	[2]	1,302	102,569
IMG/M (Aquatic metagenome subset)[2]	Metagenome & Virome	Marine & Freshwater	[3]	13,811	1,196,310
TARA	Metagenome & Virome	Marine	[4], [5]	8,611	851,642
Mediterranean contig collection	Metagenome & Virome	Marine	[6], [7],[8]	1,701	108,453
Global Oceanic Virome (GOV)[2]	Virome	Marine	[9]	1,046	109,862
IMG/VR[2]	Metagenome & Virome	Marine & Freshwater	[10]	11,837	715,672

(1) Gbp = 1,000,000 bp.
(2) Contigs from these datasets were not assembled in house but are instead the ones provided in their respective repositories.

REFERENCES

[1] Biller SJ *et al*. "Marine microbial metagenomes sampled across space and time." *Scientific Data. 2018 Sep 4;5:180176.*
[2] Silva G. Acinas *et al*. "Metabolic Architecture of the Deep Ocean Microbiome." *bioRxiv 635680; doi: https://doi.org/10.1101/635680.*
[3] Chen, I-Min A *et al*. "IMG/M v.5.0: an integrated data management and comparative analysis system for microbial genomes and microbiomes." *Nuc. acids res. 2019 Jan 8;47(D1):D666-D677.*
[4] Pesant S *et al*. "Open science resources for the discovery and analysis of Tara Oceans data." *Scientific Data. 2015 May 26;2:150023.*
[5] Alberti A *et al*. "Viral to metazoan marine plankton nucleotide sequences from the Tara Oceans expedition." *Scientific Data. 2017 Aug 1;4:170093.*
[6] Mizuno CM *et al*. "Expanding the marine virosphere using metagenomics." *PLoS Genetics. 2013;9(12):e1003987.*
[7] López-Pérez M *et al*. "Genome diversity of marine phages recovered from Mediterranean metagenomes: Size matters." *PLoS Genetics. 2017 Sep 25;13(9):e1007018.*
[8] Haro-Moreno *et al*. "Prokaryotic Population Dynamics and Viral Predation in a Marine Succession Experiment Using Metagenomics." 2019 Front. Microbiol. 2019 Dec 19;10:2926
[9] Roux S *et al*. "Ecogenomics and potential biogeochemical impacts of globally abundant ocean viruses." *Nature. 2016 Sep 29;537(7622):689-693.*
[10] Paez-Espino D *et al*. "IMG/VR v.2.0: an integrated data management and analysis system for cultivated and environmental viral genomes." *Nuc. acids res. 2019 Jan 8;47(D1):D678-D686*
