# Supplementary figures and images for: Metagenome Mining Reveals Hidden Genomic Diversity of Pelagimyophages in Aquatic Environments
Source: mSystems. 2020 Feb 18;5(1):e00905-19. doi: 10.1128/mSystems.00905-19 (PMC7029224; doi:10.1128/mSystems.00905-19)

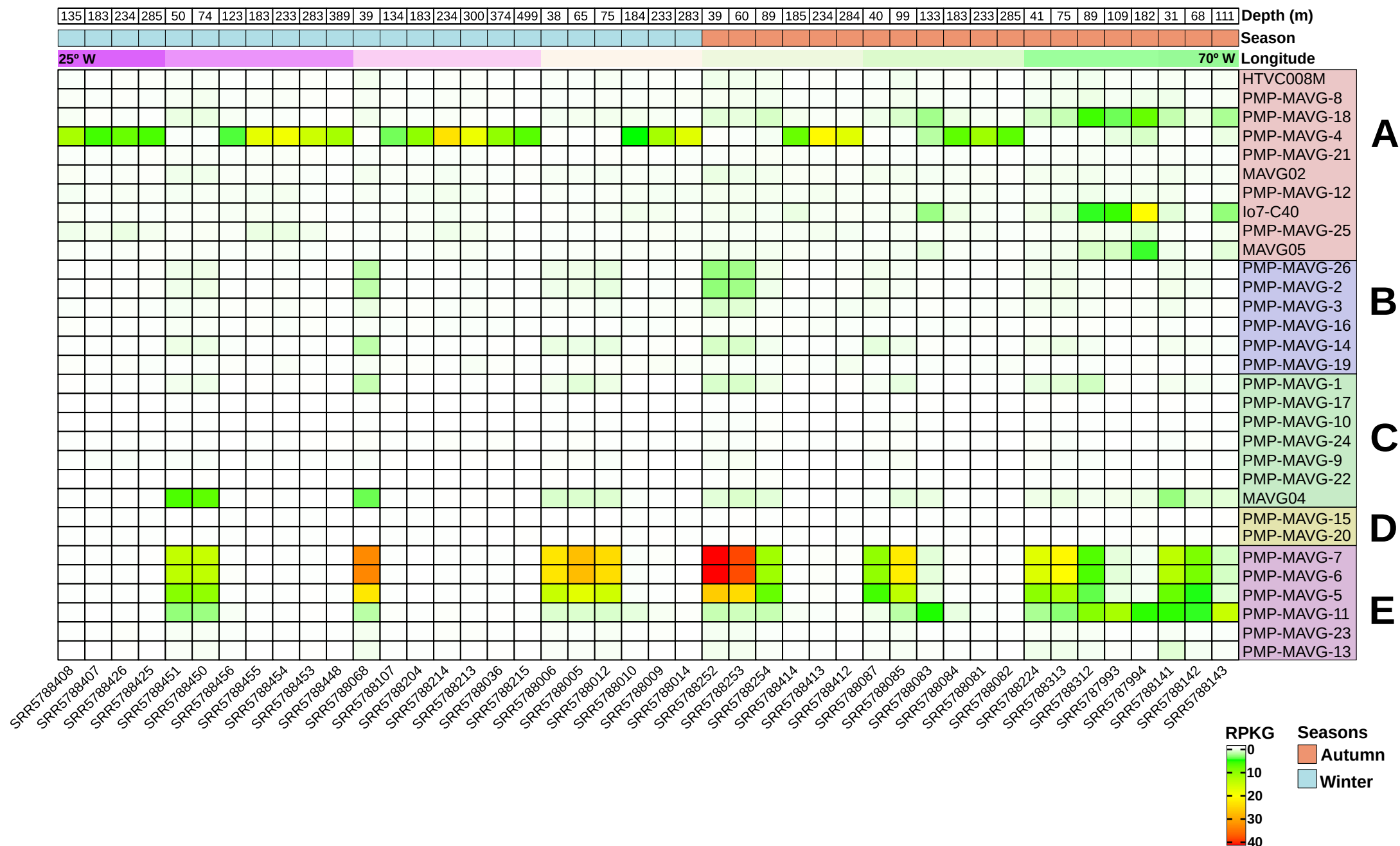

Supplement: FIG S2 [file mSystems.00905-19-sf002.pdf]
