# Supplementary material for: Metagenome Mining Reveals Hidden Genomic Diversity of Pelagimyophages in Aquatic Environments
Source: mSystems. 2020 Feb 18;5(1):e00905-19. doi: 10.1128/mSystems.00905-19 (PMC7029224; doi:10.1128/mSystems.00905-19)
Supplement: FIG S3 [file mSystems.00905-19-sf003.pdf]

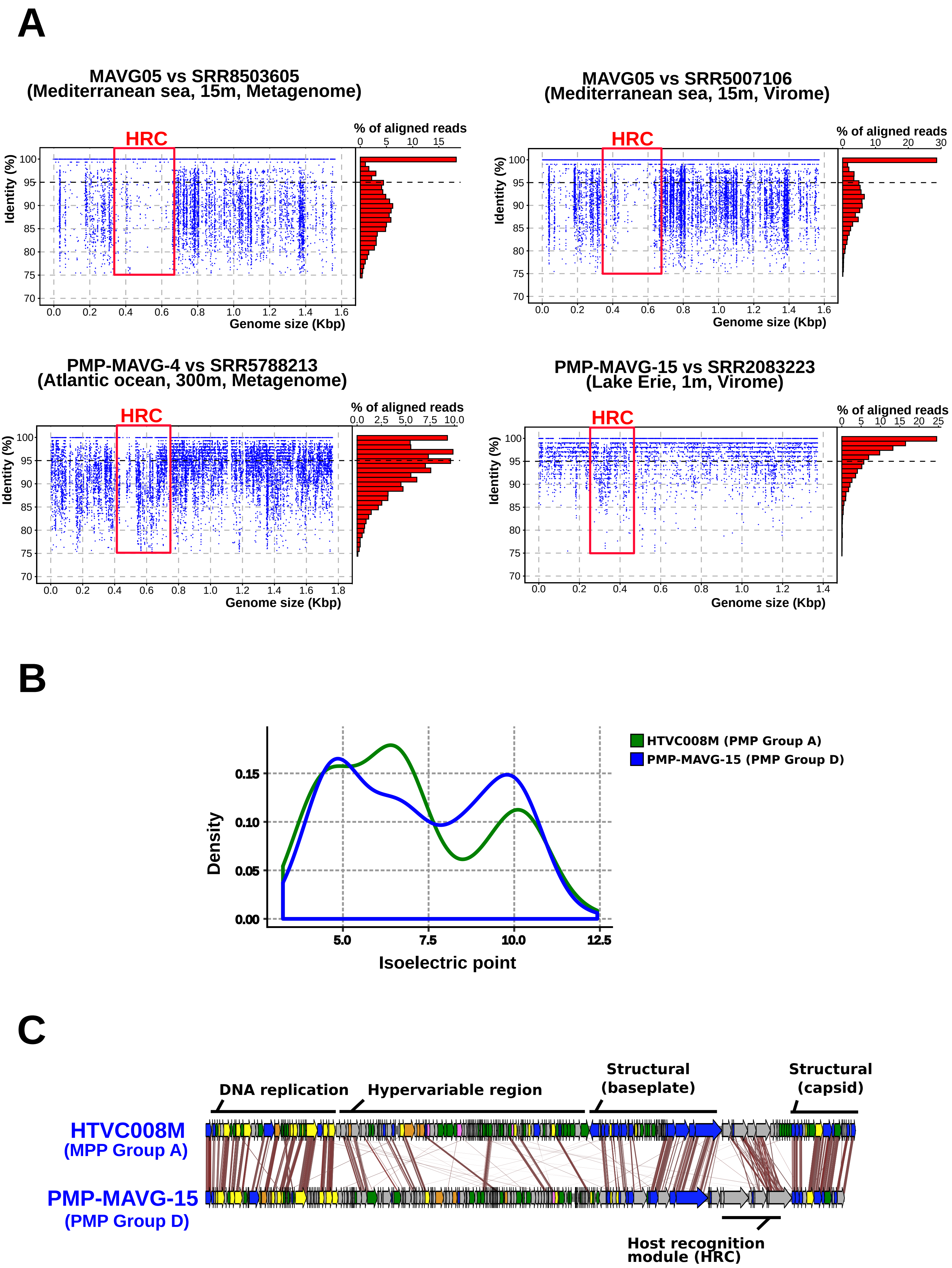
A
MAVG05 vs SRR8503605
(Mediterranean sea, 15m, Metagenome)
MAVG05 vs SRR5007106
(Mediterranean sea, 15m, Virome)
PMP-MAVG-4 vs SRR5788213
(Atlantic ocean, 300m, Metagenome)
PMP-MAVG-15 vs SRR2083223
(Lake Erie, 1m, Virome)
HRC
% of aligned reads
Identity (%)
Genome size (Kbp)
B
HTVC008M (PMP Group A)
PMP-MAVG-15 (PMP Group D)
Density
Isoelectric point
C
DNA replication
Hypervariable region
Structural (baseplate)
Structural (capsid)
HTVC008M
(MPP Group A)
PMP-MAVG-15
(PMP Group D)
Host recognition module (HRC)
