# Supplementary material for: Metagenome Mining Reveals Hidden Genomic Diversity of Pelagimyophages in Aquatic Environments
Source: mSystems. 2020 Feb 18;5(1):e00905-19. doi: 10.1128/mSystems.00905-19 (PMC7029224; doi:10.1128/mSystems.00905-19)
Supplement: FIG S4 [file mSystems.00905-19-sf004.pdf]

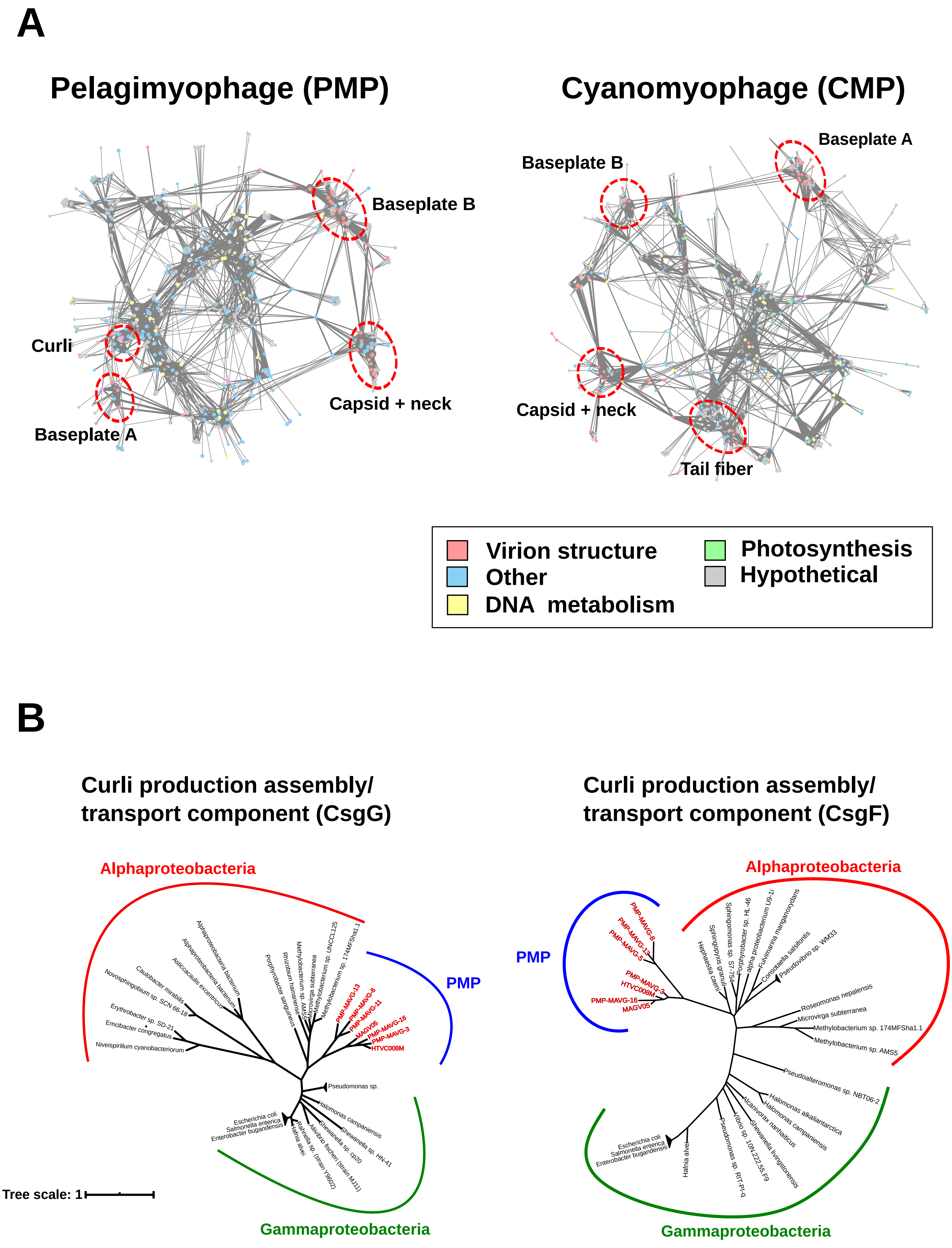
A
Pelagimyophage (PMP)
Cyanomyophage (CMP)
Baseplate B
Curli
Baseplate A
Capsid + neck
Baseplate A
Baseplate B
Capsid + neck
Tail fiber
Virion structure
Other
DNA metabolism
Photosynthesis
Hypothetical
B
Curli production assembly/
transport component (CsgG)
Curli production assembly/
transport component (CsgF)
Alphaproteobacteria
PMP
Gammaproteobacteria
Tree scale: 1
Alphaproteobacteria bacterium
Alphaproteobacteria bacterium
Asticcacaulis excentricus
Caulobacter mirabilis
Novosphingobium sp. SCN 66-18
Erythrobacter sp. SD-21
Emcibacter congregatus
Niveispirillum cyanobacteriorum
Porphyrobacter sanguineus
Rhizobium hainanense
Methylobacterium sp. AMS5
Microvirga subterranea
Methylobacterium sp. UNCCL125
Methylobacterium sp. 174MFSha1.1
PMP-MAVG-13
PMP-MAVG-8
PMP-MAVG-11
MAGV05
PMP-MAVG-16
PMP-MAVG-3
HTVC008M
Pseudomonas sp.
Halomonas campaniensis
Shewanella sp. HN-41
Shewanella sp. cp20
Aliivibrio fischeri (strain MJ11)
Rahnella sp. (strain Y9602)
Hafnia alvei
Escherichia coli
Salmonella enterica
Enterobacter bugandensis
Alphaproteobacteria
PMP
Gammaproteobacteria
PMP-MAVG-8
PMP-MAVG-13
PMP-MAVG-5
PMP-MAVG-3
HTVC008M
PMP-MAVG-16
MAGV05
Hephaestia caeni
Sphingopyxis granuli
Sphingomonas sp. S7-754
Porphyrobacter sp. HL-46
alpha proteobacterium U9-1i
Fulvimarina manganoxydans
Consotaella salsitonis
Pseudovibrio sp. WM33
Roseomonas nepalensis
Microvirga subterranea
Methylobacterium sp. 174MFSha1.1
Methylobacterium sp. AMS5
Pseudoalteromonas sp. NBT06-2
Halomonas alkaliantarctica
Halomonas campaniensis
Alcanivorax nanhaiticus
Shewanella livingstonensis
Vibrio sp. 10N.222.55.F9
Pseudomonas sp. RIT-PI-q
Hafnia alvei
Escherichia coli
Salmonella enterica
Enterobacter bugandensis
