# Supplementary material for: Metagenome Mining Reveals Hidden Genomic Diversity of Pelagimyophages in Aquatic Environments
Source: mSystems. 2020 Feb 18;5(1):e00905-19. doi: 10.1128/mSystems.00905-19 (PMC7029224; doi:10.1128/mSystems.00905-19)
Supplement: FIG S5 [file mSystems.00905-19-sf005.pdf]

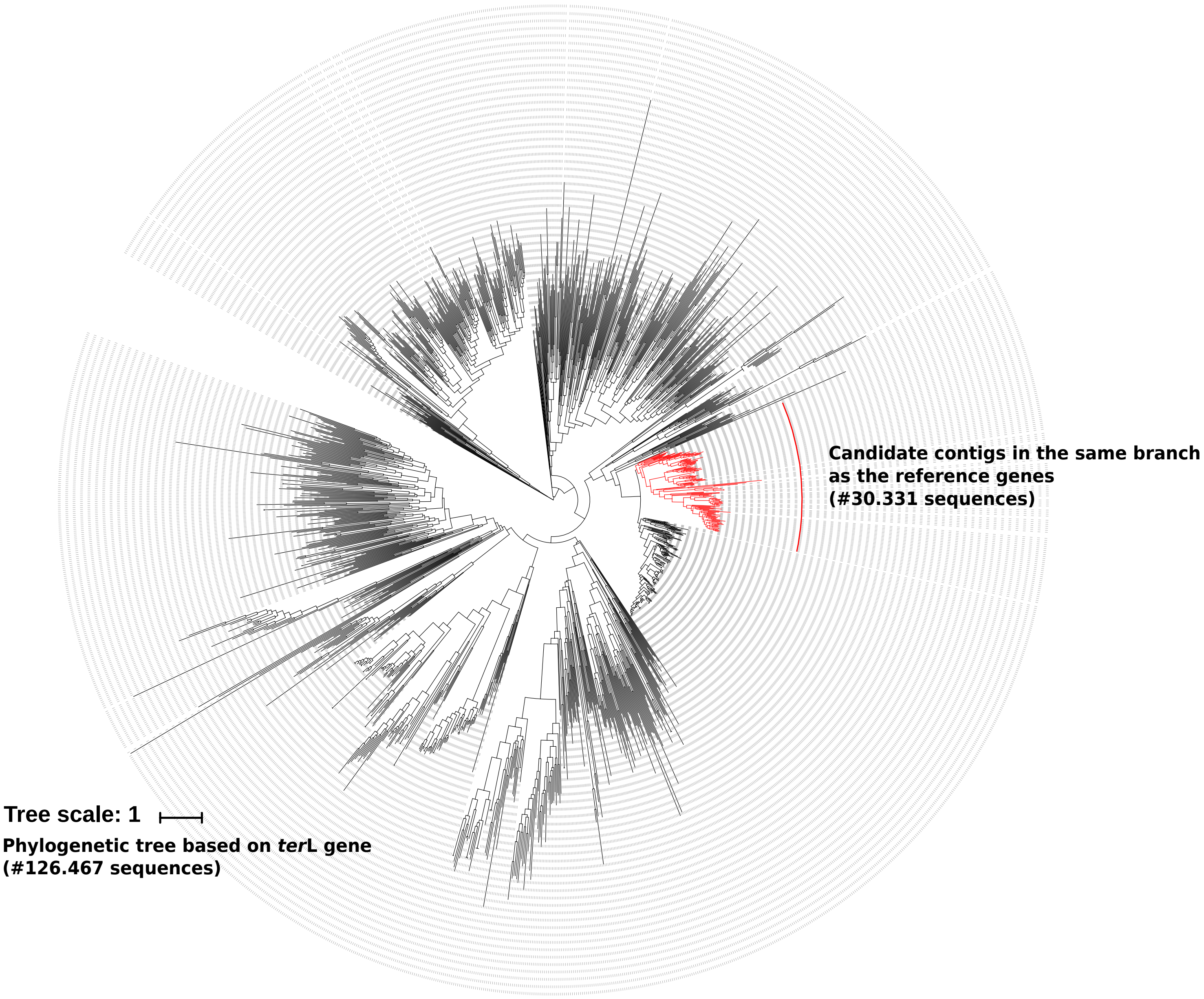
Candidate contigs in the same branch
as the reference genes
(#30.331 sequences)
Tree scale: 1
Phylogenetic tree based on *ter*L gene
(#126.467 sequences)
