# Supplementary material for: Metagenome Mining Reveals Hidden Genomic Diversity of Pelagimyophages in Aquatic Environments
Source: mSystems. 2020 Feb 18;5(1):e00905-19. doi: 10.1128/mSystems.00905-19 (PMC7029224; doi:10.1128/mSystems.00905-19)
Supplement: TABLE S4 [file mSystems.00905-19-st004.pdf]

Table S4. Protein clusters (PC) with size > 10 genes and a log fold difference of at least 1. Normalized gene counts were calculated as (Total number of genes in all genomes of the group / total genomes in the group).

Protein ID	Normalized protein count (Norm. PMP)	Normalized protein count (Norm. CMP)	Log(Norm. PMP/Norm. CMP)	Category
Putative lipase	0.37	0.00	5.00	Lipid biosynthesis
30S Ribosomal protein S21	0.83	0.00	5.00	DNA translation
5'(3')-deoxyribonuclease	0.60	0.00	5.00	Nucleotide biosynthesis
Adenine-specific RNA methyltransferase	0.37	0.00	5.00	Methyltransferase
Adenylate kinase	0.37	0.00	5.00	Nucleotide biosynthesis
Adenylate kinase adk	0.37	0.00	5.00	Regulation
Adenylate/Guanylate cyclase catalytic domain w/ CHASE2 domain	0.60	0.00	5.00	Regulation
Baseplate hub subunit gp5	0.60	0.00	5.00	Structural/Baseplate
Cell wall hydrolase	0.43	0.00	5.00	Hydrolase activity
Curlin-associated protein	0.77	0.00	5.00	Cell envelope
DNA end protector protein	0.87	0.00	5.00	DNA packaging
FeCR domain-containing protein	0.60	0.00	5.00	Substrate transport
Glycosyltransferase, family 8	1.03	0.00	5.00	LPS biosynthesis
Glycosyltransferase, family 8	0.60	0.00	5.00	LPS biosynthesis
Iron-Sulfur cluster assembly accesory protein IscA	0.53	0.00	5.00	Fe-S cluster
Iron-Sulfur cluster assembly accesory protein IscU	0.40	0.00	5.00	Fe-S cluster
Molybdenum cofactor biosynthesis protein MoaA	0.60	0.00	5.00	Radical SAM
Peptide-modifying protein SuiA/SuiB	0.57	0.00	5.00	Protein modification
Radical SAM protein + SPASM domain	0.50	0.00	5.00	Radical SAM
T4-like lysozyme	0.43	0.00	5.00	Hydrolase activity
tRNA(Ile)-lysidine synthetase	0.40	0.00	5.00	DNA translation
Type VIII secretion system (T8SS), CsgF protein	0.70	0.00	5.00	Cell envelope
Type VIII secretion system (T8SS), CsgG protein	0.70	0.00	5.00	Cell envelope
VrlC Protein	0.80	0.03	4.68	Structural/Baseplate
Cytidylyltransferase	0.63	0.03	4.34	Other
C-5 cytosine-specific DNA methylase	0.33	0.03	3.42	Methyltransferase
Peptide deformylase	0.60	0.06	3.26	Protein modification
Baseplate wedge subunit gp53	0.87	0.13	2.79	Structural/Baseplate
Ribonuclease H RnaseH	0.77	0.13	2.62	DNA replication
collagen triple repeat domain-containing protein	0.30	0.06	2.26	Cell envelope
Peroxiredoxin-like protein	0.50	0.13	2.00	Cell homeostasis
Tail protein w/ Immoglobulin fold	0.73	0.28	1.38	Structural/Fiber
SprT-like protein	0.43	0.19	1.21	Regulation
2OG-Fe(II) oxygenase superfamily protein	0.27	0.13	1.09	2OG-Fe(II) superfamily
Endonuclease YncB-like	0.40	0.81	-1.02	DNA replication
2OG-Fe(II) oxygenase superfamily protein	0.13	0.28	-1.08	2OG-Fe(II) superfamily
Glutaredoxin	0.63	1.38	-1.12	Cell homeostasis
Ferredoxin-dependent bilin reductase	0.10	0.22	-1.13	Iron metabolism
Protein structurally similar to Sm/Lsm-like RNA-binding proteins	0.77	1.75	-1.19	Regulation
Sm-like domain-containing protein	0.53	1.25	-1.23	Regulation
DNA methylase	0.10	0.25	-1.32	Methyltransferase
Tail tube protein	0.53	1.34	-1.33	Structural/Tail
2OG-Fe(II) oxygenase superfamily protein	0.13	0.34	-1.37	2OG-Fe(II) superfamily
Rnf-Nqr	0.13	0.34	-1.37	Nitrogen metabolism
2OG-Fe(II) oxygenase superfamily protein	0.70	1.88	-1.42	2OG-Fe(II) superfamily
2OG-Fe(II) oxygenase superfamily protein	0.10	0.28	-1.49	2OG-Fe(II) superfamily
Phosphate starvation-inducible protein PhoH	0.30	0.97	-1.69	Regulation
Prolyl-4 hydroxylase	0.13	0.44	-1.71	2OG-Fe(II) superfamily
Prolyl-4 hydroxylase	0.17	0.59	-1.83	2OG-Fe(II) superfamily
FAD-dependent thymidylate synthase ThyX	0.20	0.94	-2.23	Nucleotide biosynthesis
PKHD-type hydroxylase	0.07	0.31	-2.23	2OG-Fe(II) superfamily
Baseplate tail tube cap	0.13	0.66	-2.30	Structural/Tail
2OG-Fe(II) oxygenase superfamily protein	0.20	1.09	-2.45	2OG-Fe(II) superfamily
Ferrochelatase	0.07	0.41	-2.61	Iron metabolism
Baseplate wedge tail fiber connector gp9/gp10	0.07	0.50	-2.91	Structural/Baseplate
Cytitidyltransferase	0.07	0.78	-3.55	Other
6-phosphogluconate dehydrogenase	0.00	0.44	-5.00	Energy metabolism
ABC-type phosphate transport system, periplasmic component PstS	0.00	0.44	-5.00	Substrate transport
Antenna protein CpeT-like	0.00	0.75	-5.00	Photosynthesis
Baseplate wedge initiator w/ Concavalin A-like domain + YHYH domain	0.00	0.47	-5.00	Structural/Baseplate
Baseplate wedge initiator w/ YHYH domain	0.00	0.44	-5.00	Structural/Baseplate
Baseplate wedge subunit gp53	0.00	0.78	-5.00	Structural/Baseplate
cAMP phosphodiesterase	0.00	0.44	-5.00	Regulation
CP12 domain-containing protein	0.00	0.84	-5.00	Energy metabolism
DNA adenine methylase dam	0.00	0.91	-5.00	Methyltransferase
fructose-6-phosphate aldolase TalC	0.00	0.91	-5.00	Energy metabolism
Glucose 6-phosphate dehydrogenase	0.00	0.34	-5.00	Energy metabolism
High light inducible protein	0.00	0.44	-5.00	Photosynthesis
High light inducible protein	0.00	1.53	-5.00	Photosynthesis
LlaGI endonuclease	0.00	0.69	-5.00	DNA replication
Major outer membrane protein OMP1	0.00	0.97	-5.00	Cell envelope
MazE protein	0.00	0.47	-5.00	Regulation
MazG protein	0.00	0.88	-5.00	Other
PA14 domain-containing protein	0.00	0.31	-5.00	Other
Phage tail lysozyme	0.00	0.47	-5.00	Structural/Tail
Photosystem II protein, D1/D2 subunit	0.00	1.59	-5.00	Photosynthesis
Plastocyanin	0.00	0.63	-5.00	Photosynthesis
Plastoquinol terminal oxidase	0.00	0.53	-5.00	Electron transfer
S-adenosylmethionine decarboxylase speD	0.00	0.38	-5.00	Photosynthesis
Tail fiber protein	0.00	0.47	-5.00	Structural/Fiber
Tail fiber protein	0.00	0.50	-5.00	Structural/Fiber
Tail spike protein gp5	0.00	0.50	-5.00	Structural/Baseplate
VrlC protein	0.00	0.75	-5.00	Structural/Baseplate
