# Supplementary material for: Metagenome Mining Reveals Hidden Genomic Diversity of Pelagimyophages in Aquatic Environments
Source: mSystems. 2020 Feb 18;5(1):e00905-19. doi: 10.1128/mSystems.00905-19 (PMC7029224; doi:10.1128/mSystems.00905-19)
Supplement: TABLE S5 [file mSystems.00905-19-st005.pdf]

Table S5. AMGs detected in the analysed genomes

AMG Name	Function	PMP	CMP
Carbamoyltransferase	Nucleotide metabolism	X	X
CobS	Cobalamin biosynthesis	X	X
CobT	Cobalamin biosynthesis	X	X
Ferrochelatase	Heme biosynthetic pathway	X	X
Heme oxygenase	Heme degradation	X	X
Heat shock protein hsp20	Helps refold proteins in stressful conditions	X	X
Pyrophosphatase MazG	Limits the effects of mazEF in response to aminoacid starvation	X	X
PhoH	Phosphate starvation regulon Pho	X	X
Tryptophan halogenase prnA	Degradation of aromatic compounds, antibiotic biosynthesis	X	X
ABC-type phosphate transport system PstS	Part of PstABC, involved in phosphate import	X	X
PurM	Purine biosynthesis *de novo* pathway	X	X
Thioredoxin	Redox protein, plays a role in many biological processes	X	X
Ribonucleotide reductase RNA	Provides precursors for DNA synthesis (NTP → dNTP)	X	X
Prolyl-4 hydroxylase	DNA repair	X	X
Peroxiredoxin	Oxidative stress control	X	X
Acyl carrier protein acpP	Lipid biosynthesis	X	X
Rnf-Nqr	Nitrogen fixation	X	X
Peptide deformylase	Protein maturation	X	X
Ferredoxin, ISC System	Small electron carrier	X	
Iron-Sulfur cluster assembly protein IscA	Scaffold protein for Fe-S cluster biosynthesis	X	
Iron-Sulfur cluster assembly protein IscU	Scaffold protein for Fe-S cluster biosynthesis	X	
Iron-Sulfur cluster assembly protein SufE	Fe-S cluster biosynthesis	X	
asparagine synthase asnB	Synthesis of asparagine from aspartate	X	
30S ribosomal protein S21	Protein translation, recognition of Shine-dalgarno sequence	X	
50S ribosomal protein L7/L12	Protein translation, binding site for several factors	X	
Cytochrome C	Electron transport	X	
Alternative oxidase AOX	Electron transfer from reduced ubiquinol to oxygen, forming water	X	
Stearoyl-CoA desaturase (Delta-9 desaturase)	Lipid biosynthesis	X	
beta-ketoacyl-acyl-carrier-protein synthase II FabF	Lipid biosynthesis	X	
Adenylate kinase adk	Nucleotide conversion (NTP + NMP → 2 NDP)	X	
Cold shock protein CpsA	Regulation, involved in RNA folding	X	
L-lactate permease	Substrate transport	X	
TonB-dependent vitamin B12 receptor	Substrate transport	X	
Vitamin B3 transporter PnuC	Substrate transport	X	
Tripartite tricarboxylate transporter, TctA family	Substrate transport	X	
L-Aspartate-alpha-decarboxylase	CoA biosynthesis	X	
6-phosphogluconate dehydrogenase gnd	Pentose phosphate pathway		X
cAMP phosphodiesterase	Alters gene expression of genes controlled by cAMP		X
CP12	Pentose phosphate pathway		X
cpeT chromophore lyase	Site-selective attachment of cromophores		X
Glucose-6-phosphate-1 dehydrogenase zwf	Pentose phosphate pathway		X
high light inducible protein	Collect energy from protons and transfer it to photosystems		X
Plastocyanin petE	Electron transport between photosystems		X
Ferredoxin petF	Small electron carrier, part of the photsynthetic system		X
Taurine catabolism dioxygenase TauD	Part of the photosystem II reaction center		X
Photosystem II D2 protein PsbD	Part of the photosystem II reaction center		X
Plastoquinol terminal oxidase PtoX	May have a role in mantaining the reduction state of electron transport chain components		X
PurC	Purine biosynthesis *de novo* pathway		X
PurH	Purine biosynthesis *de novo* pathway		X
PurL	Purine biosynthesis *de novo* pathway		X
PurN	Purine biosynthesis *de novo* pathway		X
PurS	Purine biosynthesis *de novo* pathway		X
PyrE	Purine biosynthesis *de novo* pathway		X
S-adenosylmethionine decarboxylase proenzyme SpeD	Polyamine biosynthesis		X
Transaldolase TalC	Pentose phosphate pathway		X
Taurine catabolism dioxygenase TauD	Sulfur salvage from Taurine		X
